# Association of Knee Extension Torque, Velocity and Power with Chronological Age, Maturity Status and Physical Performance in Prepubertal Boys

**DOI:** 10.3390/children12121713

**Published:** 2025-12-18

**Authors:** Julian Alcazar, Lena Reznar, Georg Bogoevski, Peter Raidl, Gustavo Z. Schaun, Robert Csapo

**Affiliations:** 1 GENUD Toledo Research Group, Faculty of Sport Sciences, University of Castilla-La Mancha, 45071 Toledo, Spain; julian.alcazar@uclm.es; 2 CIBER on Frailty and Healthy Aging (CIBERFES), Instituto de Salud Carlos III, 28029 Madrid, Spain; 3 Grupo Mixto de Fragilidad y Envejecimiento Exitoso UCLM-SESCAM, Universidad de Castilla-La Mancha-Servicio de Salud de Castilla-La Mancha, IDISCAM, 45004 Toledo, Spain; 4 Department of Sport and Human Movement Science, Centre for Sport Science and University Sports, University of Vienna, 1150 Vienna, Austria; a01548401@unet.univie.ac.at (L.R.); bogoevski.georg@gmail.com (G.B.); peter.raidl@univie.ac.at (P.R.); gustavo.schaun@univie.ac.at (G.Z.S.)

**Keywords:** torque–velocity, force–velocity, power–velocity, countermovement jump, maturation, growth

## Abstract

**Highlights:**

**What are the main findings?**

- Absolute knee extension torque and power in prepubertal boys are strongly associated with both chronological age and, even more so, maturity status.
- High-velocity muscle function normalized to body mass is the strongest predictor of functional performance in prepubertal boys.

**What are the implications of the main finding?**

- The use of absolute values of muscle function should be discouraged for health status assessment or talent identification in prepubertal boys of the same age, since differences may arise from biological maturity.
- Muscle function normalized to body mass is the best indicator of practical, weight-bearing capabilities and functional performance in children among the studied parameters.

**Abstract:**

**Background:** This study aimed to assess the effects of age and maturity status on the knee extension torque–velocity (T-V) relationship, muscle power, and physical performance in prepubertal boys. **Methods:** This cross-sectional study included 53 prepubertal boys (9–13 years old) who underwent a physical and maturity status examination. Knee extension T-V relationship was assessed using an isokinetic device, and maximal isometric torque (T_0_), maximal unloaded velocity, maximum muscle power, and T-V slope (T-V_slope_) were derived. Physical performance was assessed using the countermovement jump (CMJ) height. Linear regression models were used, and the level of significance was set at *p* ≤ 0.05. **Results:** All torque and power parameters were significantly correlated with age (r = 0.49–0.58) and maturity status (r = 0.74–0.78) (all *p* < 0.05). No associations were found for velocity-related parameters (r = 0.20) or T-V_slope_ relative to T_0_ (r = 0.18) (both *p* > 0.05). Interestingly, the associations disappeared when muscle function parameters were normalized to body mass (all *p* > 0.05), whereas normalization to height squared only weakened the associations, which remained statistically significant (*p* < 0.05). Finally, CMJ height was most strongly associated with T-V and power parameters normalized to body mass, especially at higher contraction velocities. **Conclusions:** Torque and muscle power derived from the knee extension T-V relationship increased as a function of age and maturity status in prepubertal boys. These associations vanished upon adjustment for body mass, while normalization to height squared only weakened the correlations. Muscle function produced at faster contraction velocities and normalized to body mass emerged as the best indicator of functional muscle performance in prepubertal children among the studied neuromuscular parameters.

## 1. Introduction

Muscle function describes the ability of the neuromuscular system to produce force and power and to resist fatigue over time. Of note, muscle function is an important outcome related to physical performance, quality of life and overall health throughout the entire life cycle [1,2], including during childhood [3,4]. Muscular fitness has been positively related to brain development and academic success among children [5]. Unfortunately, the present generation of children is physically weaker than previous ones [4,6,7,8,9], less likely to adhere to physical activity recommendations, and more likely to exhibit comorbidities, activity-related injuries, and limitations in mobility [4,10]. Moreover, low levels of muscle function observed during childhood can often persist into adulthood [11,12], increasing the risk of disability and premature death later in life [13,14]. Therefore, understanding the development of muscle function during growth and maturation is of paramount importance.

In children, absolute maximal muscle strength and power increase substantially from childhood to adulthood [15,16,17,18]. Thus, 6–11-year-old children exhibit approximately 25–50% of the strength levels of young adults (11–13), whereas muscle power levels in 8–10-year-old children correspond to 30–50% of those found in young adults [18]. The improvements in muscle function observed with growth and maturation seem to be influenced by different factors, including changes in body mass, height, limb length, and muscle mass [19,20]. In 10–14-year-old children, changes in body size and mass have emerged as the strongest predictors of changes in muscle strength during growth, surpassing even the predicting power of changes in muscle cross-sectional area [20]. As a consequence, investigating the development of muscle function during maturation independently from changes in body size or mass might be key for obtaining a better understanding of this phenomenon. Such an approach promises to yield deeper insights into the specific changes occurring within the neuromuscular system.

Some earlier studies seem to support an age-associated increase in muscle function relative to body mass [21,22], whereas others showed no corresponding changes [23,24], and one investigation even presents contradictory findings [15]. These discrepancies might, in part, be related to methodological considerations. For example, normalizing muscle forces to body mass may not ideally account for changes in body dimensions. Indeed, some researchers propose that allometric scaling of muscle function to height squared is a superior approach for investigating muscle function development during growth [17,25]. Nevertheless, scaling to body mass might still be relevant from a functional and physical performance perspective, as most daily activities are weight-bearing.

More importantly, the inconsistent findings in previous studies could also be attributed to variations in the force metrics investigated. Whereas some authors reported no changes in relative isometric strength, relative strength measured during dynamic contractions increased as a function of maturation [21,22]. Of note, during dynamic contractions, force production decreases as a function of muscle shortening velocity, and different neuromuscular and anatomical factors may exert distinct influences along this force–velocity (F-V) relationship [26]. This adds another layer of complexity to the interpretation of data on the influence of biological maturation on muscle function. Although the F-V relationship is well-documented in adult populations [26], research on children and adolescents remains limited. Some studies have investigated this relationship during cycling [27,28] and a series of running sprints [29]. However, these activities require relatively high technical skills and coordination and may, therefore, not be ideally suited to assess muscle function in children. On the other hand, knee extension dynamometry has been extensively used to evaluate both isometric and dynamic muscle function across different contraction velocities in children [15]. However, fundamental parameters of the torque–velocity (T-V) relationship, such as the slope of the T-V relationship or maximum muscle power (Pmax), have not been reported.

The main goals of this investigation were to evaluate the association between the T-V relationship and muscle power of the knee extensors and age and maturation in absolute terms and normalized to body height squared and body mass, and to assess the relationship between T-V and power parameters and physical performance in prepubertal boys. We hypothesized that absolute knee extension torque and power would increase significantly with both chronological age and maturity status in prepubertal boys. Moreover, normalizing muscle function parameters to body height squared (allometric scaling) would account for the influence of body size more effectively than normalizing to body mass, potentially reducing the magnitude of the associations with age and maturity. Conversely, muscle function parameters normalized to body mass would serve as the strongest predictors of physical performance, given the weight-bearing nature of this kind of task.

## 2. Methods

### 2.1. Study Design

To investigate the association between muscle function, chronological age, maturity status and physical performance in prepubertal boys, a cross-sectional study was conducted between September 2022 and June 2023. The sample included boys aged 9 to 14 years, who were recruited from schools located in the city of Vienna through personal contact. Muscle function was comprehensively evaluated by the assessment of the T-V and power relationship of the knee extensors. Thus, the participants were required to attend the laboratory to complete the anthropometric, maturity, physical and muscle function assessments. An a priori power analysis was performed for simple linear regression (t-test on a single regression coefficient) using G*Power 3.1. Assuming a two-tailed alpha level of 0.05, a desired power of 0.80, and a medium effect size (f^2^ = 0.15), a required sample size of n = 55 was determined.

### 2.2. Subjects

A total of 55 boys agreed to participate in this investigation (9 years old, n = 1; 10 years old, n = 12; 11 years old, n = 11; 12 years old, n = 19; 13 years old, n = 11; 14 years old, n = 1). Participants were included if they were below their estimated age at peak height velocity (PHV), which was determined as described below. None of the participants had medical contraindications preventing them from completing the physical tests. Written informed consent was obtained from both the children and their parents. The present study was approved by the ethics committee of the University of Vienna (vote number 00824) and conducted in accordance with the Helsinki Declaration.

### 2.3. Procedures

#### 2.3.1. Anthropometrics

For anthropometric measurements, the participants were barefoot and wore light clothing. Standing and sitting heights were measured (±0.01 m) using a stadiometer (Seca 213, Seca GmbH & Co. KG., Hamburg, Germany), and body mass was evaluated (±0.1 kg) with a digital scale (Seca 877, Seca GmbH & Co. KG., Hamburg, Germany). Body mass index (BMI) was then calculated as body mass divided by height squared (kg·m^−2^).

#### 2.3.2. Maturity Status

Maturity status was determined by estimating the age at PHV using the non-invasive method proposed by Mirwald and colleagues [30]. Briefly, this method estimates the maturity offset, defined as the time before or after the age at which PHV is reached, based on standing and sitting height, body mass and chronological age. Only boys with a negative maturity offset (i.e., before age at PHV) were considered for this study.

#### 2.3.3. Torque–Velocity–Power Relationship

Unilateral knee extension muscle function of the dominant leg was assessed using an isokinetic dynamometer (Isomed 2000, D. & R. Ferstl GmbH, Hemau, Germany). Subjects were seated upright on the dynamometer chair with the axis of the dynamometer aligned with the axis of their knee joint. The participants’ shoulders, waist, thigh, and lower leg were secured with straps, and their arms were kept crossed over the chest during all the trials. After familiarization with the test procedures and completion of a specific warm-up, several isometric and isokinetic trials were conducted. Peak torque values were recorded during 3–4 s maximal isometric contractions at 70° of knee flexion (full extension = 0°). Three trials were performed, and the highest peak torque (Nm) value was defined as the maximal voluntary isometric contraction torque. Peak torque was further recorded during maximal isokinetic contractions performed at 60 deg·s^−1^, 120 deg·s^−1^ and 180 deg·s^−1^. Participants performed two trials at each angular velocity and the highest peak torque values (Nm) were used for further analysis. To minimize fatigue-related bias, the sequence in which tests at varying velocities were conducted was randomized [31]. All participants were instructed to complete each repetition as fast and strong as possible through the whole range of movement (from 90° to full extension). Strong verbal encouragement was provided to the participants during each trial, and adequate resting periods were allowed between trials (30 s) and between loading conditions (120 s).

To derive the T-V relationship parameters, custom-made software was used to fit the torque and velocity data acquired during both the isometric and isokinetic contractions using a linear T-V equation [26]:

$$V=\frac{T-T_{0}}{{TV}_{slope}}$$

where V is angular velocity, T is torque, T_0_ is the estimated maximum isometric torque, and T-V_slope_ is the slope of the linear T-V relationship. T-V_slope_ denotes the decrement in torque as a function of velocity and was reported in absolute values and also as a percentage of T_0_ to denote the intrinsic ability to keep torque levels with increasing angular velocities. In addition, the estimated maximal unloaded velocity (V_0_) was calculated as the intercept of the velocity axis. Then, power was calculated as the product of torque and angular velocity, and maximum muscle power (P_max_), optimal torque (T_opt_) and optimal velocity (V_opt_) were identified at the apex of the P-V relationship. To optimize the evaluation of the T-V relationship, and in consideration of basic physiological principles, the software incorporated automatized instructions for the selection of suitable torque and angular velocity data [32]: maximum isometric torque values lower than any of the recorded dynamic torque values were excluded; dynamic trials with lower torque values for a given angular velocity compared to a faster contraction as well as dynamic trials exhibiting lower power for a corresponding angular velocity compared to both slower and faster contractions were discarded. Isometric or dynamic trials visually displaying reduced torque compared to the values expected based on the remaining trials were only removed if the difference between measured and estimated values exceeded 10% after excluding those trials. Participants were included in the analysis only if the fit of their T-V data yielded a coefficient of determination (R^2^) of at least 0.90. Application of this criterion resulted in the exclusion of 2 participants, so 53 participants were considered for further analyses. Finally, the average R^2^ was 0.99 ± 0.01 and the standard error of the estimate (SEE) was 3.07 ± 4.43 Nm. The results are reported both in absolute terms and after normalization to body mass and height squared, respectively.

#### 2.3.4. Countermovement Jump

Participants performed three countermovement (CMJ) jumps interspersed by 60 s of rest on a force plate (9286BA, Kistler Instrumente AG, Winterthur, Switzerland). Participants were instructed to stand with their hands resting on their hips and feet hip-width apart and to jump as high as possible following the cue “ready, set, go!”. Moreover, they were asked to land with both feet hip-width apart on the force plate and to avoid flexing their knees before landing. Peak force, velocity and power were extracted from the data recorded by the force plate. In addition, take-off velocity was used to estimate CMJ height [33]. The trial corresponding to the highest CMJ height was considered for further analyses. The CMJ results are reported in absolute terms and normalized to body mass and height squared.

### 2.4. Statistical Analysis

Data are presented as mean ± standard deviation unless otherwise stated. The associations between the different dependent variables with chronological age and maturity offset (independent variables) were assessed using linear regression models. The relationship between the different knee extension muscle function parameters and physical performance assessed through CMJ height was also investigated by linear regression models. All statistical analyses were carried out with SPSS version 24 (SPSS Inc., Chicago, IL, USA) and the level of significance was set at α = 0.05.

## 3. Results

The study participants’ main characteristics are presented in Table 1.

**Figure 1:**
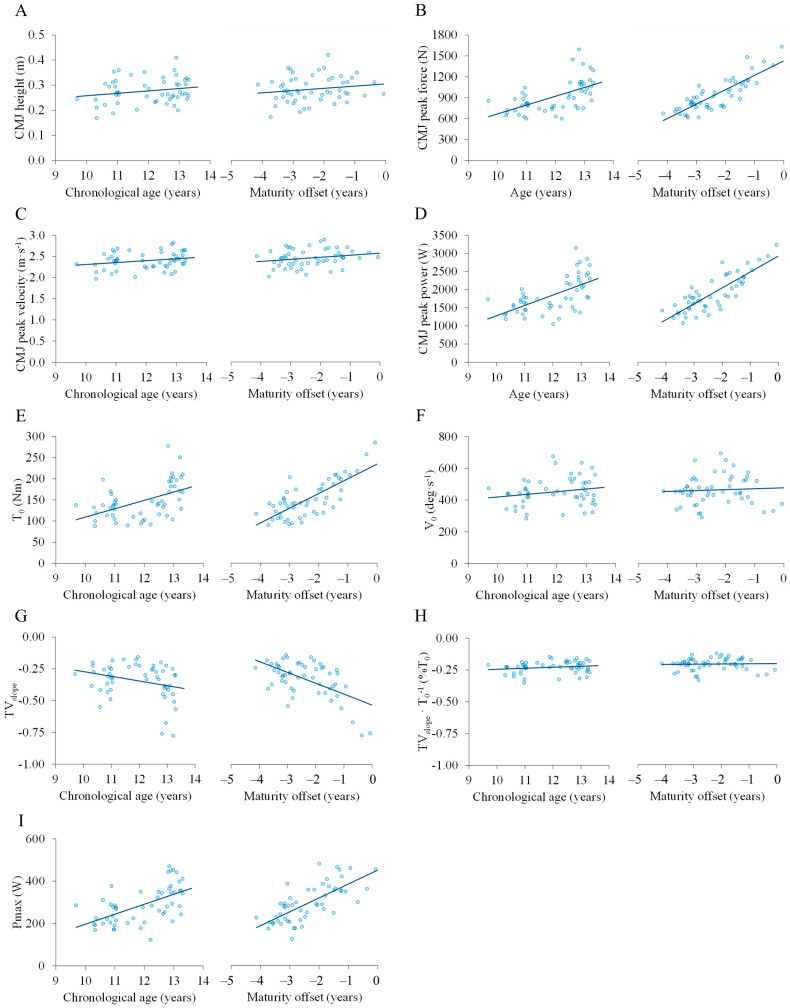
Associations of the main outcomes obtained from the countermovement jump test and the torque–velocity relationship with chronological age and maturity status: (**A**) CMJ height. (**B**) CMJ peak force. (**C**) CMJ peak velocity. (**D**) CMJ peak power. (**E**) T0. (**F**) V0. (**G**) TVslope. (**H**) TVslope normalized to T0. (**I**) Pmax. Open symbols represent individual data, and continuous lines represent regression equations. Note: CMJ, countermovement jump. T_0_, estimated maximal isometric torque. V_0_, estimated maximum unloaded contraction velocity. TV_slope_, slope of the linear torque–velocity relationship. P_max_, maximum muscle power.

The associations between the main outcomes obtained from the T-V relationship and the CMJ test with chronological age and maturity offset are illustrated in Figure 1. When expressed in absolute terms (Table 2), significant positive associations were observed between CMJ peak force and peak power with both age and maturity offset (all *p* < 0.001), while no associations were found for CMJ peak velocity or CMJ height (both *p* > 0.05). For the T-V relationship-derived measures, significant positive associations were found between all torque and power outcomes and age and maturity offset (all *p* < 0.001). In addition, a significant negative association was observed between T-V_slope_ and both age and maturity offset (both *p* ≤ 0.041). In contrast, no association was found within T-V_slope_ when torque data was normalized to T_0_ (*p* > 0.05).

When normalized to height squared (Table 3), there was a significant positive association between CMJ peak force and power and both age and maturity offset (all *p* ≤ 0.021). Regarding the T-V and power outcomes, a significant positive association with age was observed for torque and power at 120 and 180 deg·s^−1^, as well as P_max_ (all *p* < 0.034). The maturity offset was positively correlated to torque and power at 0–180 deg·s^−1^, P_max_ and T_opt_, and negatively associated with T-V_slope_ (all *p* ≤ 0.012). No other significant associations were found (all *p* > 0.05).

On the other hand, when the outcome measures were normalized to body mass (Table 4), only T-V_slope_ was found to be significantly and positively associated with age and maturity offset (both *p* < 0.001). No significant association was noted for any of the other torque and power parameters extracted from either the CMJ or T-V relationship (all *p* > 0.05).

Finally, all associations between CMJ height and the different outcomes are reported in Table 5. Briefly, CMJ height was significantly associated with torque at 180 deg·s^−1^ (r = 0.29, *p* = 0.037), V_0_ (r = 0.32, *p* = 0.021), power at 180 deg·s^−1^ (r = 0.29, *p* = 0.037), P_max_ (r = 0.35, *p* = 0.010), V_opt_ (r = 0.32, *p* = 0.021), relative torque at 0–180 deg·s^−1^ (r = 0.28 to 0.47, *p* ≤ 0.046), relative power at 60–180 deg·s^−1^ (r = 0.35 to 0.47, *p* ≤ 0.011), relative P_max_ (r = 0.52, *p* < 0.001), relative T_opt_ (r = 0.28, *p* = 0.048), allometric torque and power at 180 deg·s^−1^ (both r = 0.30, *p* = 0.034), and allometric P_max_ (r = 0.37, *p* = 0.006). No associations between CMJ height and T-V_slope_ or any other outcomes were found (all *p* > 0.05).

## 4. Discussion

The present study investigated the relationship of muscle function with age and maturity status, as well as the associations between muscle function parameters and physical performance, in prepubertal boys. The main findings were that (i) CMJ force and power and knee extension torque and power increased with increasing age and maturity status; (ii) accounting for body size (height squared) generally reduced, and in some cases eliminated, the association between muscle function and age, while the associations with maturity status remained statistically significant, albeit weaker; (iii) no associations were found between muscle function normalized to body mass and age or maturity status, with the exception of the slope of the T-V relationship; and (iv) physical performance assessed through CMJ height was more strongly correlated with knee extension muscle function at higher contraction velocities (≥180 deg·s^−1^), especially when normalized to body mass.

According to our findings, both maximal isometric and dynamic torques increase at a rate of approximately 13–15% per year in boys aged 9 to 13 years, while maximum muscle power increases by approximately 16% per year. Of note, these results agree with those reported in longitudinal studies showing that knee extension torque produced at different contraction velocities increase by 14–18% per year during a 5-year follow-up in 11-year-old boys [24]. Regarding the structure of the T-V relationship, the slope became more negative with age and maturity status. However, when normalized to T_0_ it remained constant. This normalization provides a relative indicator of how increasing contraction velocities affect the ability to produce torque and power. In young, middle-aged and older adults, the aging process has been found to progressively impair the ability to produce torque and power under increasing contraction velocities [32]. Our findings denote that, unlike in adult cohorts, this hallmark of aging remains stable in prepubertal boys during maturation. Additionally, as per our correlation coefficients, muscle function appeared more strongly related to maturity status than to chronological age. Maturation is a process by which individuals progress towards a mature biological state [34], and as such, it is accompanied by changes in hormonal levels [35], body composition [36], anthropometrics [34], and specific changes in the neuromuscular system [37], among others, that can ultimately lead to substantial improvements in the rate at which muscle function increases. Maturation has been proven to be an important factor for both talent identification [38] and injury risk detection [39].

The cross-sectional area and mean fiber size of the vastus lateralis muscle of 25-year-old adults have been reported to be twice as large as those from 5-year-old children, whereas the number of muscle fibers remained constant [36]. Moreover, changes in body size with growth provoke an inevitable increase in muscle length and number of sarcomeres in series. Thus, the vastus lateralis muscle exhibits an increase in muscle fascicle length (but not pennation angle) with age in prepubertal children [40]. These changes in muscle size could partially explain not only the increase in maximal isometric torque but also the increase in torque production at any given contraction velocity. Further, joint moment arms also increase with growth, favoring greater torque production with increasing biological maturity [41].

As torque augments as a function of muscle area, the theory of geometric similarity suggests that torque improves as a function of body height [25]. Thus, the use of allometric scaling of forces or torques to height squared has been suggested as an adequate approach to control for the effect of muscle size. When the results of the present study were normalized to height squared, the strength of the associations between muscle function and chronological age or maturity status was approximately reduced by half, and in some cases for chronological age, the statistical significance disappeared. This may indicate that other changes occurring in the neuromuscular system, apart from skeletal muscle hypertrophy, may produce muscle function gains in 9- to 13-year-old boys. For instance, the proportion of type I fibers in the vastus lateralis muscle has been found to decrease from ~65% in 5-year-old children to ~50% in young adults in their 20s [36]. Consequently, a corresponding increase in the proportion of type II muscle fibers is expected, together with specific hypertrophy in type IIA muscle fibers [42]. In addition, animal studies suggest that ATPase activity, which determines single muscle fiber shortening velocity, is lower in pre-pubertal compared to young adult rats [43]. In humans, twitch relaxation time has been found to be higher in children compared to young adults, which would predispose children to slower muscle contractile speeds [44]. Moreover, musculo-tendinous stiffness also increases during growth, thereby improving the ability to transmit forces rapidly from muscles to bones [45]. Additional changes, such as the maturation of the pyramidal system and myelination of motor neurons [34,37], as well as improved central activation [34], muscle fiber conduction velocity [46], and ability to recruit high-threshold motor units [47], support the existence of relevant neuromuscular changes that explain the muscle size-independent increase in muscle function with maturation [48,49].

Another, more traditional approach to normalizing measures of muscle function is using body mass. This method may be preferable since most activities of daily living and sports activities performed by children involve supporting their own body mass. Our findings do not indicate an increase in torque and power normalized to body mass in boys aged 9 to 13 years. Pääsuke et al. [21] found significant differences in dynamic knee extension torque normalized to body mass between 11- and 16-year-old boys, whereas Holm et al. [22] observed longitudinal increments in boys only after the age of 13 years. Hence, it is likely that increases in muscle function relative to body mass occur during the so-called adolescent spurt, which starts approximately at the age at which PHV is attained (~14 years old in boys) [24,25]. On the other hand, the best correlates of physical performance in the current study were the production of knee extension torque and power normalized to body mass, which confirms the relevance of these measures in terms of functional muscle performance. Specifically, the association was strengthened as a function of contraction velocity. In this sense, the lack of association between muscle function normalized to body mass and age or maturity status could explain why no relationship was found between CMJ height and age or maturity status either.

There are some limitations in the current study that should be considered. The cross-sectional design impedes the establishment of cause–effect relationships. However, for the main study findings, there is a body of literature that supports the direct relationship between gains in muscle function and physiological changes occurring with age and maturation in prepubertal boys, as has been discussed. The present study did not include girls due to difficulties in recruitment and, therefore, further experiments exploring the association between knee extension muscle function with age and maturity status in girls are required. Despite this, the current literature supports the notion that there are no significant differences in muscle function between boys and girls in the age range of the participants included in our investigation [15,35], the exception being muscle function normalized to body mass, which tends to be greater in boys in some studies [23]. Moreover, knee extension muscle function was directly measured at velocities ranging from 0 to 180°·s^−1^. Of note, linear T-V models might not be adequate to estimate T-V parameters in the range of low torque and high velocity values (i.e., below 45% of T_0_) [50,51]. Nevertheless, the main conclusions of the current study were based on the observable part of the T-V relationship, and the use of linear models has been found to be adequate for the assessed portion of the T-V relationship. Finally, in contrast to the a priori power analysis, 2 participants had to be excluded following data inspection, resulting in a final sample size of 53 participants. This minor reduction is unlikely to have meaningfully affected the ability to detect the primary associations of interest, particularly given that many of the observed relationships were of moderate to large magnitude.

## 5. Conclusions

In prepubertal boys, absolute knee extension torque and power are strongly determined by biological maturity rather than chronological age. While normalizing to height squared reveals contributions from neuromuscular maturation, normalizing to body mass effectively eliminates the confounding influence of somatic growth. Crucially, high-velocity muscle power normalized to body mass emerges as the superior indicator of functional, weight-bearing performance. Therefore, for valid health and performance assessment in this population, practitioners should prioritize mass-normalized metrics at high contraction velocities to avoid the biases inherent in absolute measures.

## Figures and Tables

**Table 1:** Main characteristics of the study participants (n = 53).

	Mean	±	SD	Range
Age (years)	12.0	±	1.1	9.7–13.3
Body mass (kg)	43.8	±	10.3	28.7–78.8
Height (m)	1.54	±	0.10	1.39–1.76
BMI (kg·m^−2^)	18.1	±	2.4	13.8–26.2
Sitting height (m)	0.72	±	0.05	0.61–0.86
Maturity offset (years)	−2.38	±	0.95	−4.14–0.07
Age at PHV (years)	14.3	±	0.6	12.9–15.6

Note: SD, standard deviation. BMI, body mass index. PHV, peak height velocity.

**Table 2:** Relationship of physical performance and muscle function with age and maturity offset.

	Mean	±	SD	Chronological Age			Maturity Offset		
				r	(95% CI)	*p* Value	r	(95% CI)	*p* Value
*CMJ*									
Height (m)	0.28	±	0.05	0.20	(−0.08, 0.45)	0.152	0.16	(−0.11, 0.42)	0.246
Peak force (N)	925.3	±	232.4	**0.58**	**(0.37, 0.74)**	**<0.001**	**0.84**	**(0.74, 0.91)**	**<0.001**
Peak velocity (m·s^−1^)	2.41	±	0.21	0.24	(−0.04, 0.48)	0.090	0.23	(−0.05, 0.47)	0.106
Peak power (W)	1862.2	±	505.4	**0.61**	**(0.40, 0.76)**	**<0.001**	**0.83**	**(0.72, 0.90)**	**<0.001**
*TVP relationship*									
T_0_ (Nm)	149.0	±	43.3	**0.49**	**(0.26, 0.67)**	**<0.001**	**0.76**	**(0.61, 0.85)**	**<0.001**
T_60_ (Nm)	128.3	±	36.4	**0.52**	**(0.30, 0.70)**	**<0.001**	**0.77**	**(0.64, 0.86)**	**<0.001**
T_120_ (Nm)	107.6	±	30.2	**0.56**	**(0.34, 0.72)**	**<0.001**	**0.78**	**(0.64, 0.87)**	**<0.001**
T_180_ (Nm)	86.9	±	25.2	**0.58**	**(0.36, 0.73)**	**<0.001**	**0.74**	**(0.59, 0.84)**	**<0.001**
V_0_ (deg·s^−1^)	453.6	±	88.2	0.20	(−0.07, 0.45)	0.149	0.06	(−0.21, 0.33)	0.667
T-V_slope_	−0.35	±	0.14	**−0.28**	**(−0.51, −0.01)**	**0.041**	**−0.57**	**(−0.73, −0.36)**	**<0.001**
T-V_slope_ (%T_0_)	−0.23	±	0.05	0.18	(0.09, 0.43)	0.195	0.03	(−0.24, 0.30)	0.829
P_60_ (W)	134.4	±	38.1	**0.52**	**(0.30, 0.70)**	**<0.001**	**0.77**	**(0.64, 0.86)**	**<0.001**
P_120_ (W)	225.4	±	63.2	**0.56**	**(0.34, 0.72)**	**<0.001**	**0.78**	**(0.64, 0.87)**	**<0.001**
P_180_ (W)	273.0	±	79.3	**0.58**	**(0.36, 0.73)**	**<0.001**	**0.74**	**(0.59, 0.84)**	**<0.001**
P_max_ (W)	291.9	±	90.1	**0.58**	**(0.37, 0.74)**	**<0.001**	**0.71**	**(0.55, 0.82)**	**<0.001**
T_opt_ (Nm)	74.6	±	21.6	**0.49**	**(0.26, 0.67)**	**<0.001**	**0.76**	**(0.62, 0.85)**	**<0.001**
V_opt_ (deg·s^−1^)	226.8	±	44.1	0.20	(−0.07, 0.45)	0.149	0.06	(−0.21, 0.33)	0.667

Note: SD, standard deviation. CMJ, countermovement jump. TVP, torque–velocity–power. T_0_, estimated maximal isometric torque. T_60_, T_120_, T_180_, torque at 60, 120 and 180 deg·s^−1^, respectively. V_0_, estimated maximum unloaded contraction velocity. T-V_slope_, slope of the linear torque–velocity relationship. P_60_, P_120_, P_180_, power at 60, 120 and 180 deg·s^−1^, respectively. P_max_, maximum muscle power. T_opt_, optimal torque. V_opt_, optimal velocity. Bold values indicate *p* < 0.05.

**Table 3:** Relationship of physical performance and muscle function normalized to height squared with age and maturity offset.

	Mean	±	SD	Chronological Age			Maturity Offset		
				r	(95% CI)	*p* Value	r	(95% CI)	*p* Value
*CMJ*									
Peak force (N·m^−2^)	380.9	±	60.7	**0.32**	**(0.05, 0.54)**	**0.021**	**0.57**	**(0.35, 0.73)**	**<0.001**
Peak power (W·m^−2^)	765.1	±	138.5	**0.38**	**(0.12, 0.59)**	**0.005**	**0.57**	**(0.36, 0.73)**	**<0.001**
*T-V relationship*									
T_0_ (Nm·m^−2^)	61.4	±	12.7	0.21	(−0.06, 0.46)	0.126	**0.46**	**(0.22, 0.65)**	**<0.001**
T_60_ (Nm·m^−2^)	52.8	±	10.5	0.25	(−0.02, 0.49	0.072	**0.48**	**(0.24, 0.66)**	**<0.001**
T_120_ (Nm·m^−2^)	44.3	±	8.8	**0.29**	**(0.02, 0.52)**	**0.034**	**0.48**	**(0.24, 0.67)**	**<0.001**
T_180_ (Nm·m^−2^)	35.8	±	7.6	**0.33**	**(0.06, 0.55)**	**0.017**	**0.44**	**(0.20, 0.64)**	**<0.001**
T-V_slope_ (·m^−2^)	−0.13	±	0.04	−0.12	(−0.37, 0.16)	0.413	**−0.34**	**(−0.56, −0.08)**	**0.012**
P_60_ (W·m^−2^)	55.3	±	11.0	0.25	(−0.02, 0.49)	0.072	**0.48**	**(0.24, 0.66)**	**<0.001**
P_120_ (W·m^−2^)	94.6	±	16.4	**0.39**	**(0.13, 0.59)**	**0.004**	**0.46**	**(0.22, 0.65)**	**<0.001**
P_180_ (W·m^−2^)	112.4	±	24.0	**0.33**	**(0.06, 0.55)**	**0.017**	**0.44**	**(0.20, 0.64)**	**<0.001**
P_max_ (W·m^−2^)	120.1	±	28.1	**0.35**	**(0.09, 0.57)**	**0.009**	**0.43**	**(0.18, 0.63)**	**0.001**
T_opt_ (Nm·m^−2^)	30.7	±	6.3	0.21	(−0.06, 0.46)	0.125	**0.46**	**(0.22, 0.65)**	**<0.001**

*Note:* SD, standard deviation. CMJ, countermovement jump. TVP, torque–velocity–power. T_0_, estimated maximal isometric torque. T_60_, T_120_, T_180_, torque at 60, 120 and 180 deg·s^−1^, respectively. T-V_slope_, slope of the linear torque–velocity relationship. P_60_, P_120_, P_180_, power at 60, 120 and 180 deg·s^−1^, respectively. P_max_, maximum muscle power. T_opt_, optimal torque. Bold values indicate *p* < 0.05.

**Table 4:** Relationship of physical performance and muscle function normalized to body mass with age and maturity offset.

	Mean	±	SD	Chronological Age			Maturity Offset		
				r	(95% CI)	*p* Value	r	(95% CI)	*p* Value
*CMJ*									
Peak force (N·kg^−1^)	20.9	±	2.1	0.04	(−0.23, 0.31)	0.759	0.04	(−0.23, 0.31)	0.769
Peak power (W·kg^−1^)	42.1	±	6.0	0.14	(−0.14, 0.40)	0.312	0.12	(−0.16, 0.38)	0.409
*TVP relationship*									
T_0_ (Nm·kg^−1^)	3.37	±	0.51	−0.03	(−0.30, 0.25)	0.851	0.05	(−0.22, 0.32)	0.707
T_60_ (Nm·kg^−1^)	2.91	±	0.42	0.01	(−0.26, 0.28)	0.918	0.06	(−0.21, 0.33)	0.653
T_120_ (Nm·kg^−1^)	2.44	±	0.36	0.07	(−0.20, 0.34)	0.608	0.07	(−0.20, 0.34)	0.602
T_180_ (Nm·kg^−1^)	1.97	±	0.34	0.14	(−0.14, 0.39)	0.337	0.08	(−0.20, 0.34)	0.562
T-V_slope_ (·kg^−1^)	−0.005	±	0.002	**0.56**	**(0.34, 0.72)**	**<0.001**	**0.65**	**(0.46, 0.78)**	**<0.001**
P_60_ (W·kg^−1^)	3.04	±	0.44	0.01	(−0.26, 0.28)	0.918	0.06	(−0.21, 0.33)	0.653
P_120_ (W·kg^−1^)	5.11	±	0.76	0.07	(−0.20, 0.34)	0.608	0.07	(−0.20, 0.34)	0.602
P_180_ (W·kg^−1^)	6.19	±	1.08	0.14	(−0.14, 0.39)	0.337	0.08	(−0.20, 0.34)	0.562
P_max_ (W·kg^−1^)	6.61	±	1.33	0.19	(−0.09, 0.43)	0.185	0.10	(−0.17, 0.36)	0.463
T_opt_ (Nm·kg^−1^)	1.69	±	0.26	−0.03	(−0.30, 0.25)	0.849	0.05	(−0.22, 0.32)	0.715

Note: SD, standard deviation. CMJ, countermovement jump. TVP, torque–velocity–power. T_0_, estimated maximal isometric torque. T_60_, T_120_, T_180_, torque at 60, 120 and 180 deg·s^−1^, respectively. T-V_slope_, slope of the linear torque–velocity relationship. P_60_, P_120_, P_180_, power at 60, 120 and 180 deg·s^−1^, respectively. P_max_, maximum muscle power. T_opt_, optimal torque. Bold values indicate *p* < 0.05.

**Table 5:** Associations between countermovement jump height and the different outcomes obtained from the torque–velocity–power relationship.

	Absolute			Normalized to Body Mass			Normalized to Height Squared		
	r	(95% CI)	*p* Value	r	(95% CI)	*p* Value	r	(95% CI)	*p* Value
T_0_	0.15	(−0.13, 0.41)	0.282	**0.28**	**(0.01, 0.51)**	**0.046**	0.11	(−0.17, 0.37)	0.448
T_60_	0.19	(−0.09, 0.44)	0.183	**0.35**	**(0.09, 0.57)**	**0.011**	0.16	(−0.12, 0.41)	0.267
T_120_	0.23	(−0.04, 0.48)	0.095	**0.43**	**(0.18, 0.63)**	**0.002**	0.22	(−0.05, 0.47)	0.113
T_180_	**0.29**	**(0.02, 0.52)**	**0.037**	**0.47**	**(0.23, 0.66)**	**<0.001**	**0.30**	**(0.02, 0.53)**	**0.034**
V_0_	**0.32**	**(0.05, 0.54)**	**0.021**						
T-V_slope_	0.02	(−0.25, 0.29)	0.878	0.18	(−0.10, 0.43)	0.196	0.09	(−0.19, 0.35)	0.531
T-V_slope_ (%T_0_)	0.27	(−0.01, 0.50)	0.054						
P_60_	0.19	(−0.09, 0.44)	0.183	**0.35**	**(0.09, 0.57)**	**0.011**	0.16	(−0.12, 0.41)	0.267
P_120_	0.23	(−0.04, 0.48)	0.095	**0.43**	**(0.18, 0.63)**	**0.002**	0.09	(−0.19, 0.36)	0.522
P_180_	**0.29**	**(0.02, 0.52)**	**0.037**	**0.47**	**(0.23, 0.66)**	**<0.001**	**0.30**	**(0.02, 0.53)**	**0.034**
P_max_	**0.35**	**(0.09, 0.57)**	**0.010**	**0.52**	**(0.29, 0.70)**	**<0.001**	**0.37**	**(0.11, 0.59)**	**0.006**
T_opt_	0.15	(−0.13, 0.41)	0.282	**0.28**	**(0.01, 0.51)**	**0.048**	0.11	(−0.17, 0.37)	0.458
V_opt_	**0.32**	**(0.05, 0.54)**	**0.021**						

Note: T_0_, estimated maximal isometric torque. T_60_, T_120_, T_180_, torque at 60, 120 and 180 deg·s^−1^, respectively. V_0_, estimated maximum unloaded contraction velocity. T-V_slope_, slope of the linear torque–velocity relationship. P_60_, P_120_, P_180_, power at 60, 120 and 180 deg·s^−1^, respectively. P_max_, maximum muscle power. T_opt_, optimal torque. V_opt_, optimal velocity. Bold values indicate *p* < 0.05.

## Data Availability

All data are available upon reasonable request to the corresponding author.
